# Passive Measurement of Three Optical Beacon Coordinates Using a Simultaneous Method

**DOI:** 10.3390/s21155235

**Published:** 2021-08-02

**Authors:** Jiri Nemecek, Martin Polasek

**Affiliations:** Department of Aviation Technology, Faculty of Military Technology, University of Defence, 66210 Brno, Czech Republic; martin.polasek@unob.cz

**Keywords:** optical beacon, camera, passive position measurement, simultaneous method, LED, measurement errors

## Abstract

Among other things, passive methods based on the processing of images of feature points or beacons captured by an image sensor are used to measure the relative position of objects. At least two cameras usually have to be used to obtain the required information, or the cameras are combined with other sensors working on different physical principles. This paper describes the principle of passively measuring three position coordinates of an optical beacon using a simultaneous method and presents the results of corresponding experimental tests. The beacon is represented by an artificial geometric structure, consisting of several semiconductor light sources. The sources are suitably arranged to allow, all from one camera, passive measurement of the distance, two position angles, the azimuth, and the beacon elevation. The mathematical model of this method consists of working equations containing measured coordinates, geometric parameters of the beacon, and geometric parameters of the beacon image captured by the camera. All the results of these experimental tests are presented.

## 1. Introduction

The article describes how to measure a position of an artificially created object, an optical beacon (hereinafter *beacon*), in relation to one measuring camera (hereinafter *camera*). The below presented simultaneous analytical method is one of the passive methods for measuring an object’s relative position.

### 1.1. Problem Statement

The measurement of object position is an important problem that is solved in numerous areas of human activities. Many different instruments and methods have been developed for outdoor and indoor positioning.

Passive methods using images of some objects of interest constitute one group of methods of measuring the relative position of objects. Some analytical methods are theoretically developed, experimentally verified, and practically used as well [1,2,3,4,5,6]. There are some studies that use stereo vision for measuring position [7,8] or a combination of one camera and another sensor such as a sonar or laser range finder [9,10]. Cameras are also used along with inertial navigation systems [7,11]. This task can be solved using neural networks as well [12,13].

The proposed passive method does not require complex and extensive instrumentation. It needs one camera as the only sensor. On the other hand, the necessary condition for the application of this method is the use of a specific system of feature points, which have to be defined on a surface of the object of interest. The feature points do not have to transmit a specific optical signal. They can create an artificial beacon, which serves as a reference object for a measuring unit equipped with an image sensor. The paper presents a positioning system of this type. Due to the design of the beacon, spans of measured angular coordinates are constrained. However, the beacon layout can be modified so that a span of measured values of position angles is significantly extended.

### 1.2. Literature Review

A number of different techniques have been developed for indoor positioning systems. Some of these methods are based on evaluating either the time of arrival (TOA) or the time difference of arrival (TDOA) of an optical signal to the receiver; others are based on the received signal strength (RSS) depending on the receiver position [14,15,16,17], and there are also methods based on a receiver position determination according to the angle of arrival (AOA) [18,19,20,21,22,23,24]. Time methods use modulated signal travel time between several reference sources and a receiver. If the source irradiance spatial pattern is known, e.g., light-emitting diode (LED) [25], certain angle methods use the dependence of the received signal strength on the incident angle and on the angle between the normal radiation source and the direction to the receiver. Position angles can be determined also without knowledge of the radiation spatial distribution [16]. Other methods utilize the dependence of a position or of individual source image sizes in the plane of the image sensor [22,26]. The AOA method is also used in a quadrant angular diversity aperture (QADA) receiver, which is equipped with a quadrant photodiode (PD) and an aperture shifted from the PD plane by a small distance. The QADA receiver can be combined with an image sensor [27].

Measuring systems consist of one or several radiation sources and receivers. The radiation of the fixed reference sources located in a room, for example on the ceiling, can be modulated in accordance with the applied method. The sources’ coordinates have to be known. The predominant, but not the only, radiation source type is LED semiconductor. Photodiodes [14,16] or image sensors [18,19,20,21] or both together are used to detect radiation on the receiver side. Optoelectronic receivers can be combined with magnetic field sensors [23], accelerometers [14,16], gyroscopes, or inertial navigation systems, depending on the method [24]. Active optoelectronic sensors, e.g., lidars, are utilized too [22]. Utilization of the scale-invariant feature transform (SIFT) method is described in [18]. Indoor positioning systems also use artificial neural networks (see [18,24,28,29]). Feature points can have an arbitrary nature. In order to be identifiable, they need to be sufficiently contrasting. No specific radiation pattern needs to be assumed to determine the beacon position. The measuring error of the position coordinates is in the order of centimeters when the radiation sources and the receivers are at a distance in the order of meters.

### 1.3. Principle of the Simultaneous Analytical Method

**Figure 1:**
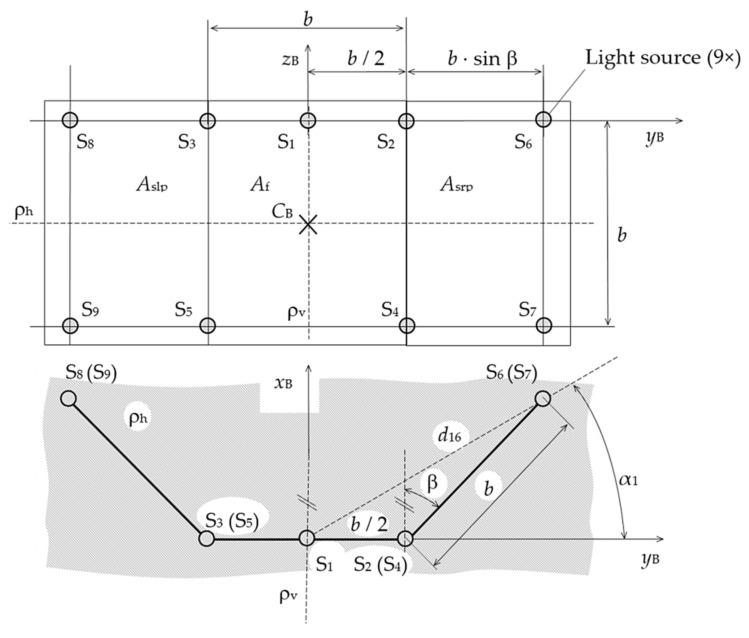
Beacon layout [30,31].

The solution presented in this article is based on the utilization of one camera. We use the specific object, beacon, and the simultaneous measurement of individual quantities. This method provides the possibility of measuring three positional coordinates if the transverse axis of the beacon and the transverse axis of the camera lie in parallel planes. The diagram of the beacon made for experimental test purposes is shown in Figure 1 [30,31], where *A*_f_ is the beacon front wall; *A*_slp_ and *A*_srp_ are projections of the left and right beacon side walls, respectively; *b*, *d*_16_, α_1_, and β are parameters of the beacon; *C*_B_ is the center of the beacon; S_1_ to S_9_ are light sources (S_1_ is the reference light source); *S*_1_*x*_B_*y*_B_*z*_B_ is the beacon coordinate system; and ρ_h_ and ρ_v_ are the horizontal and vertical planes, respectively.

**Figure 2:**
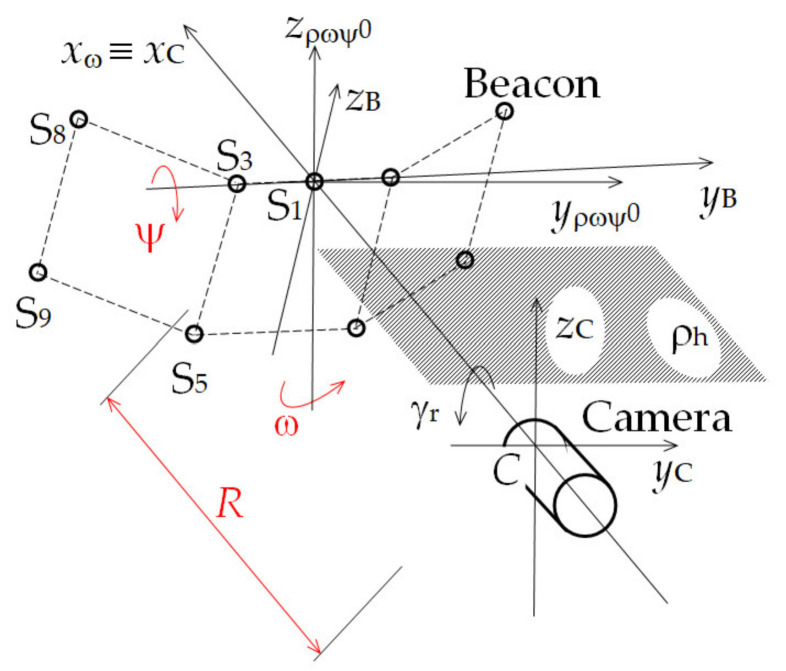
Diagram of the measuring system.

The beacon enables satisfactory measurement of the distance between the beacon and the camera *R* (hereinafter *beacon distance*), the beacon position angle in a horizontal plane ω (hereinafter *azimuth*), and the beacon elevation angle ψ (hereinafter *elevation*). The angles are created by rotating the beacon around its transverse axes *z*_ρ__ωψ0_ and *y*_B_, respectively (see Figure 2), where *Cx*_C_*y*_C_*z*_C_ is the camera coordinate system, *S*_1_*x*_ω_*y*_ρ__ωψ0_*z*_ρ__ωψ0_ is the reference coordinate system, and γ_r_ is a mutual tilt between the camera and the beacon. Nine semiconductor light sources of LED type (hereinafter *diodes*) represent sources of the optical signals, which are captured by the camera. According to the mutual position of the diodes on the beacon, and according to the diodes’ mutual position in their images in the plane of the camera sensor, the desired quantities can be then determined. The diodes create three square walls of the beacon with side *b*: a front wall *A*_f_ and two side walls, left *A*_sl_ and right *A*_sr_. The main parameters of the beacon are the base *b* and the beacon opening angle β. The side wall projection sizes *A*_slp_ and *A*_srp_ depend on these parameters. 

The evaluation of the position of the beacon diode images was done manually. The position coordinates were calculated and the measurement results were processed on a personal computer. Nowadays, the practical use of this method in real time is not possible. To do this, it is necessary to make a purpose-built automatic measuring device and to create the necessary software similar to those described in [32], where a smaller version of the optical beacon is used. A basic unsolved task is the measurement of the real range of the system for which the position coordinates reach their limits determined by the permissible errors.

The aim of the paper is to explain the method principle and to present the test results obtained during the experiments verifying the suitability of the simultaneous analytical method for measuring the beacon distance, azimuth, and elevation. The rest of the paper is organized as follows: The simultaneous analytical method is described in Section 2. Section 3 provides experimental test results. Sources of errors are characterized in Section 4. Section 5 details ways of mathematical model adjustment. Section 6 presents the conclusion. 

## 2. Description of the Simultaneous Analytical Method

The simultaneous analytical method for measuring two position coordinates was published in [30,31]. In these publications, we use working equations enabling measurement of the beacon distance *R* and the azimuth ω. These equations represent a mathematical model of this method. The method principle consists in a calculation of several so-called functional beacon distances (hereinafter *functional distances*) based on known distances between the two selected diodes and the measured distances between images of these diodes that were captured by the camera. Measuring two coordinates, as presented in [30,31], enables the determination of the position of an object only in the 2D plane. The only position coordinate on which the working equations depend is the azimuth ω. Functional distances were calculated for the so-called functional diodes S_2_, S_3_, S_6_, and S_8_ according to projections onto the *y*_ρ__ψω0_ axis. The problem can be solved only with the use of the functional distances for the diodes S_6_ and S_8_. When measuring the beacon position in the plane, the functional distance equation for the diode S_8_, for example, is as follows [30,31]:

(1) $$R_{81}=f\cdot \left[\frac{d_{16}\cdot \cos\left(\alpha_{1}-\omega \right)}{b_{81}^{\prime }}-1\right]-d_{16}\cdot \sin\left(\alpha_{1}-\omega \right),$$

where *b*^’^_81_ is the distance between the image of the S_1_ reference diode and the image of the S_8_ functional diode in the plane of the camera detector and *d*_16_ and α_1_ are the parameters of the beacon (see Figure 1).

For the measurement of the position of an object in 3D, the mathematical model had to be extended by at least one functional distance of one of the functional diodes S_4_, S_5_, S_7_, and S_9_, which lie on the lower line of the beacon. The working equations had to be adjusted also to include projections onto the z_ρ__ψω0_ axis, if they exist, which depend on both azimuth ω and elevation ψ. At least three functional distances have to be used as working equations. Such equations have to be chosen that their manifestation for at least one pair of functional diodes is opposite for both position angles. The chosen calculation uses parallel projections of the distances, between the reference diode S_1_ and all eight functional diodes S_2_ to S_9,_ onto appropriate axes of the reference rectangular coordinate system. Its axis *x*ω is identical to the optical axis of the camera *x*_C_. Then, for one particular functional diode, its functional distance for the provided azimuth and elevation is the calculated distance between the reference diode and the camera. The working equations are derived from the lens equations of the projections of the distances between the relevant diodes. 

The angles ω and ψ are unknown variables that are computed as the numbers for which the deviation between the beacon functional distances, for the individual functional diodes, is a minimum. In other words, we are looking for the minimum of the root mean square (rms) of the difference *D*_rms_ between the individual functional distances and the mean of these functional distances. When the minimum is reached, the set azimuth, the set elevation, and the mean of the calculated functional distances are equal to the desired measured variables ω_m_, ψ_m_, and *R*_m_, respectively. The following formulas, i.e., the working equations for the functional diodes *S*_3_, *S*_5_, *S*_8_, and *S*_9_ represent the mathematical model of this method:

(2) $$R_{31}=f\cdot \left(\frac{PS_{3\rho \omega \psi 0}^{}}{b_{31y}^{}}-1\right)-PS_{3x\omega }^{},$$

where $PS_{3x\omega }^{}=-0.5\cdot b\cdot \sin\omega$ and $PS_{3\rho \omega \psi 0}^{}=0.5\cdot b\cdot \cos\left(-\omega \right)$;

(3) $$R_{51}=f\cdot \left[\frac{\sqrt{{\left(PS_{5\rho \omega \psi 0}^{y}\right)}^{2}+{\left(PS_{5\rho \omega \psi 0}^{z}\right)}^{2}}}{\sqrt{b_{51y}^{2}+b_{51z}^{2}}}-1\right]-PS_{5x\omega }^{},$$

where $PS_{5x\omega }^{}=-0.5\cdot b\cdot \sin{\omega +\delta }_{x\psi 5}$, $PS_{5\rho \omega \psi 0}^{y}=0.5\cdot b\cdot \cos{\omega +\delta }_{y\psi 5}$, $PS_{5\rho \omega \psi 0}^{z}=b\cdot \cos\psi$, $\delta_{x\psi 5}=b\cdot \sin\psi \cdot \cos\omega$, and $\delta_{y\psi 5}=b\cdot \sin\psi \cdot \sin\omega$;

(4) $$R_{81}=f\cdot \left[\frac{\sqrt{{\left(PS_{8\rho \omega \psi 0}^{y}\right)}^{2}+{\left(PS_{8\rho \omega \psi 0}^{z}\right)}^{2}}}{\sqrt{b_{81y}^{2}+b_{81z}^{2}}}-1\right]-PS_{8x\omega }^{},$$

where $PS_{8x\omega }^{}=d_{16}\cdot \left[\sin\left(\alpha_{1}-\omega \right)-\sin\alpha_{1}\cdot \cos\omega \cdot \left(1-\cos\psi \right)\right]$,$PS_{8\rho \omega \psi 0}^{y}=d_{16}\cdot \left[\cos\left(\alpha_{1}-\omega \right)-\sin\alpha_{1}\cdot \sin\omega \cdot \left(1-\cos\psi \right)\right]$, and $PS_{8\rho \omega \psi 0}^{z}=d_{16}\cdot \sin\alpha_{1}\cdot \sin\psi$;

(5) $$R_{91}=f\cdot \left[\frac{\sqrt{{\left(PS_{9\rho \omega \psi 0}^{y}\right)}^{2}+{\left(PS_{9\rho \omega \psi 0}^{z}\right)}^{2}}}{\sqrt{b_{91y}^{2}+b_{91z}^{2}}}-1\right]-PS_{9x\omega }^{},$$

where $PS_{9x\omega }^{}=d_{16}\cdot \sin\left(\alpha_{1}-\omega \right)+\delta_{x\psi 9}$, $PS_{9\rho \omega \psi 0}^{y}=d_{16}\cdot \cos\left(\alpha_{1}-\omega \right)+\delta_{y\psi 9}$, $PS_{9\rho \omega \psi 0}^{z}=d_{17\rho v}\cdot \cos\left(\alpha_{2\rho v}+\psi \right)$, $\delta_{x\psi 9}=\left(a_{\psi 9}-a_{0}\right)\cdot \cos\omega$, $\delta_{y\psi 9}=\left(a_{\psi 9}-a_{0}\right)\cdot \sin\omega$, $a_{\psi 9}=d_{17\rho v}\cdot \sin\left(\alpha_{2\rho v}+\psi \right)$, $a_{0}=d_{16}\cdot \sin\alpha_{1}$, $d_{17\rho v}=\sqrt{b^{2}+{\left(d_{16}\cdot \sin\alpha_{1}\right)}^{2}}$, and $\alpha_{2\rho v}=\arctan\frac{d_{16}\cdot \sin\alpha_{1}}{b}$. 

The above equations were derived assuming that the *y*_B_ beacon axis and *y*_C_ camera axis are parallel to the horizontal plane ρ_h_ (see Figure 2). Their mutual roll angle γ_r_, which is created by the beacon or the camera rotation around the camera optical axis, was considered to be zero. The variables expressed with (2) to (5) are the functional distances *R*_i1_ for diodes S_i_, where *i* = 3, 5, 8, 9, and reference diode S_1_. They are the beacon distances computed from the projections of the distances *PS*_i_ (between the reference diode S_1_ and the functional diodes) onto the individual axes of the reference rectangular coordinate system and from the projections of the image distances *b*_i1_ of these diodes onto transverse axes *y*_C_ and *z*_C_ of the camera coordinate system *Cx*_C_*y*_C_*z*_C_. The reference coordinate system is parallel with the camera coordinate system. The axis *x*_ω_ of the reference coordinate system and the axis *x*_C_ of the camera coordinate system are identical with the camera optical axis. The origin of the reference system is at the intersection of the axis *x*_ω_ and the beacon front wall *A*_f_ (in the place of the reference diode S_1_); the axes *y*_ρ__ωψ0_ and *z*_ρ__ωψ0_ lie in the plane ρ_ωψ0_, which is identical with the beacon front wall *A*_f_ for ω = ψ = 0°. In this case, *y*_ρωψ0_ ≡ *y*_B_ and *z*_ρωψ0_ ≡ *z*_B_. The azimuth and the elevation are formed by the beacon rotation around the axis *z*_ρ__ωψ0_ and *y*_B_, respectively. Parameters *d*_16_ and α_1_ are derived from the beacon base *b* and the beacon opening angle β (see Figure 1). The working equations for the rest of the functional diodes are performed analogically. The elements *PS*_i*x*ω_ are corrections of the deviation between the object distances of the functional diodes and the measured beacon distance. 

For all the functional diodes, the rms of the functional distance differences *D*_rms_ (m) is as follows:

(6) $$D_{\mathrm{rms}}=\sqrt{\frac{1}{8}\cdot \sum_{i=2}^{9}D_{i1}^{2}},$$

where *D*_i1_ (m) is the functional distance difference *D*_i1_ (m) for the diode pair S_i_ and S_1_. It is given by the formula

(7) $$D_{i1}=R_{i1}-R_{M},$$

where $R_{M}$ (m) is the mean of the calculated functional distances. This mean is expressed by the following formula:

(8) $$R_{M}=\frac{\sum_{i=2}^{9}R_{i1}}{8}.$$

Changing the azimuth and the elevation in the working equations of the mathematical model leads to changes in the mean of the calculated distances and the rms of the distance differences. Assuming that the input position angles are equal to the true azimuth and the elevation, the beacon parameters are set exactly according to the selected values, and the camera has a high resolution, we could theoretically expect the rms of the distance differences to be practically zero.

## 3. Experimental Test Results

**Figure 3:**
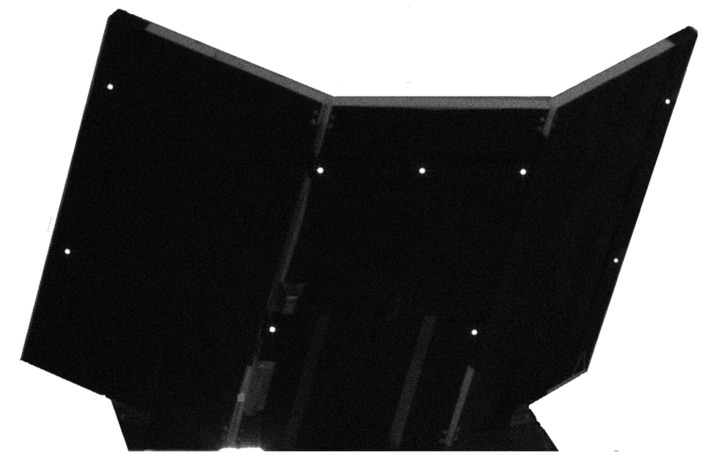
Beacon, check test, ω_n_ = 20°, ψ_n_ = 35°.

Three basic experimental tests and several check tests were performed. Their aim was to verify the functionality of the created mathematical model and the suitability of the simultaneous analytical method for measuring the optical beacon distance, azimuth, and elevation. The beacon base *b* and its opening angle β were equal to 470 mm and 56.2°, respectively (see Figure 1). The beacon was placed on a positioning mechanism, which was put on the Thorlabs RBB12A rotation stage. The MOTICAM 1080 camera was used, having two lenses with different focal lengths. Figure 3 shows beacon in the nominal position of ω_n_ = 20° and ψ_n_ = 35° during check test. 

The rotation stage was used to set the actual beacon azimuth. The conventionally true azimuth ω_0_ was measured using a scale of the actual rotation stage with the error of 2.5′. The azimuth nominal values ω_n_ were selected, around which the actual azimuth was set. The positioning mechanism was used to set the beacon elevation to nominal discrete values ψ_n_ in the range from 0 to 35° with the step of 5°. The conventionally true elevation values ψ_0_ were measured using the Fortum model 4780200 inclinometer with the error of ±0.1°. The conventionally true beacon distances *R*_0_ were measured using the Leica Disto D510 laser distance meter with the error of 1 mm. The nominal azimuth ω_n_ and elevation ψ_n_ were introduced to mark the groups of the conventionally true position angles as the azimuth was set randomly and, due to the random elevation of the relatively loose beacon fixation, the actual beacon elevation differed from the elevation set by the positioning mechanism.

**Figure 4:**
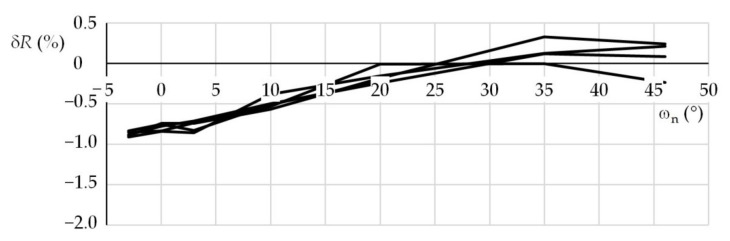
Beacon distance percentage errors; first test, ψ_n_ = 20°.

**Figure 5:**
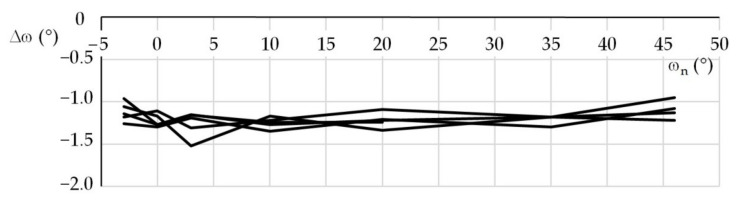
Azimuth errors; first test, ψ_n_ = 20°.

**Figure 6:**
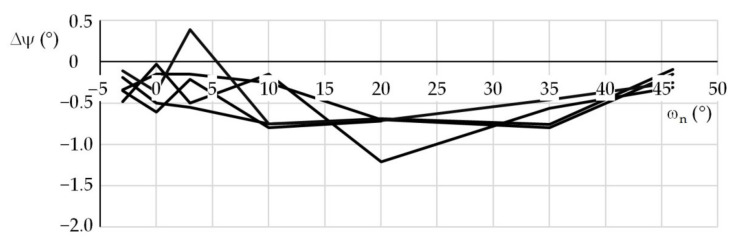
Elevation errors; first test, ψ_n_ = 20°.

The first experiment was performed using a lens with a focal length *f*_L_ = 120 mm. The beacon distance *R*_0_ was 46,520 mm. The nominal azimuth ω_n_ (°) and elevation ψ_n_ (°) were {−3, 0, 3, 10, 20, 35, 46} and {0, 5, 20, 35}, respectively. For the individual nominal elevations, the azimuths were set around their nominal values and the beacon image was recorded. Five test series were performed for every elevation. Obtained results were used for statistical processing of errors. The second and third tests were performed with the same beacon, however, using a lens with a focal length *f*_L_ = 25 mm. The beacon distances were 13,460 and 46,728 mm, respectively. The setup of these tests was the same as for the first test. One hundred forty photos were taken for each distance and focal length; in total, 420 photos were taken. Table 1 shows examples of distance *R*_m_, azimuth ω_m_, and elevation ψ_m_ that were measured during the first experiment in the first and second test series, for the nominal elevation of 20°. For comparison, the conventionally true values *R*_0_, ω_0_, and ψ_0_ are also shown. Figure 4, Figure 5 and Figure 6 show the distance percentage errors δ*R* and the position angles errors Δω and Δψ for the same test and in all the five test series. 

The distance measurements from the first test were as follows: For the nominal elevation ψ_n_ = 0°, the mean of the measured distances $\bar{R}$ was 46,675 mm. For the nominal elevations of 5, 20, and 35°, the means of the measured distances were 46,668, 46,620, and 46,643 mm, respectively. Corresponding sample standard deviations *s_R_* were 100, 127, 197, and 355 mm, respectively. 

Results from the second experiment were as follows: For ψ_n_ (°) ∈ {0, 5, 20, 35}, the means of the measured distances were 13,513, 13,550, 13,518, and 13,499 mm. The corresponding sample standard deviations *s_R_* (mm) ∈ {45, 45, 64, 105}. 

From the measured distances in the third experiment, for the same above-mentioned nominal elevation, we yielded $\bar{R}\left(\mathrm{mm}\right)\;\{47,000,\;46,933,\;46,933,\;46,925\}$ and *s_R_* (mm) ∈ {189, 129, 220, 374}. The presented values were determined from the test results of all five series.

The means and sample standard deviations of the distance percentage errors as well as azimuth and elevation errors, gained from the results of all three experiments, are listed in Table 2, Table 3 and Table 4. The measurement error frequency of these individual quantities is expressed in percentage from the total number of measurements for the individual nominal elevations. The intervals of the absolute distance percentage errors |δ*R*| are 0.0 to 0.1%, 0.0 to 0.5%, and 0.0 to 1.0%. The spans of the absolute azimuth errors |Δω|and absolute elevation errors |Δψ|are 0.0 to 0.5°, 0.0 to 1.0°, and 0.0 to 2.0°.

The measured azimuth and elevation means and their sample standard deviations are not presented as their conventionally true values mostly differed from the nominal values and were not the same even in the individual test series.

For azimuth, the error frequency was determined for the error deviations between the individual tests and the mean error of the particular test series. The reason for selecting these deviations was the beacon’s random default position in relation to the rotation stage and in relation to the support base in the azimuth. These random positions manifested themselves as a component of systematic errors. The default position differed for the individual elevations as a result of the necessary manipulation with the beacon. Thus, the relatively large nonzero mean errors were proportional mainly to the magnitudes of the unspecified default position of the beacon.

The elevation error frequency was evaluated analogously to the distance errors, according to the differences between the conventionally true and the measured values. In fact, the unspecified beacon elevation did not shift the measurement errors too much outside the selected intervals that were expected for the frequency.

## 4. Sources of Errors

The measurement errors of the simultaneous analytical method had the following basic causes:

- Differences between the actual beacon and camera parameters and the parameters that were entered into the mathematical model;
- Inaccuracies in determining the diode picture coordinates;
- Aberration of the camera lens;
- Inaccuracies in determining the beacon and camera mutual position.

### 4.1. Differences between the Actual Beacon and Camera Parameters and the Mathematical Model Parameters

The beacon base, the opening angle, and the camera focal length are all used in the beacon mathematical model. If the actual real values differ from the values entered into the model, methodological measurement errors occur. In general, these errors occur in all three measured coordinates. This fact worsens the measurement accuracy; however, on the other hand, it enables adjusting the measuring system with its optimal beacon parameters for which the accuracy indicator is the best. By modifying the mathematical model parameters, all the measured quantities can be affected. Especially, manipulation with the focal length of the camera lens is important in the mathematical model. It enables optimizing the beacon distance measurement accuracy, and at the same time, it does not influence the beacon measured angles.

### 4.2. Inaccuracies in Determining the Diode Picture Coordinates

The influence of the pixel number error (pixel coordinate error) was tested on the mathematical model, based on the fifth series of the first experiment results, with the beacon elevation of 5°. The azimuth of 10° was selected. 

For one beacon diode, the number of pixels was increased by one or by two for the *y*_C_ coordinate. Subsequently, the corresponding distance, azimuth, and elevation were determined. The influence of the pixel coordinates was tested for all nine diodes. The change of the measured beacon position was always measured only for the *y*_C_ pixel coordinate of one diode. The gained results are provided in Table 5.

Changes of the position angles Δω_p1_, Δψ_p1_ and Δω_p2_, Δψ_p2_ are the differences between the original azimuth and elevation and the new azimuth and elevation for the increased pixel coordinates *y*_C0_ + 1 and *y*_C0_ + 2, respectively, where *y*_C0_ is the original pixel coordinate. A change of the pixel coordinate just in one direction can affect all three measured quantities. The distance deviations are not presented in Table 5 as they were only in order of hundredths of a percent. The maximum of the distance error changes was about 0.15%. 

It is clear from the table that most deviations for both angles were in order of tenths of a degree. Their maximum magnitude was 0.3° for the change by one pixel and 0.5° for the change by two pixels. The deviations were zero in some cases. They occurred not only when the pixel coordinate was increased by one pixel, but also when the pixel coordinate was increased by two pixels. The nonzero deviations for two pixels were in most cases larger than deviations for one pixel. They were never smaller.

When the pixel coordinates changed for several diodes, the measured position angles changed by more than one degree. This was verified by the pixel coordinates being assessed by two persons independent of each other. The deviations of the determined pixel number were observable at both the *y*_C_ coordinates and the *z*_C_ coordinates. The biggest difference found between the measured position angles was 1.5°. It is clear that the method is sensitive to the accuracy of determining the pixel coordinates.

### 4.3. Lens Aberration

In general, camera lens aberrations can be the cause of measurement errors. The distortion of all the used lenses was experimentally verified. The distortions were not measurable. Other lens aberrations were compensated manually. For these reasons, the influence of the lens aberrations on the measurement accuracy was not considered.

### 4.4. Inaccuracies in Determining Mutual Position of the Beacon and the Camera

The azimuth measurement results were burdened with some systematic errors due to the fact that some components of the azimuth were not determined and included in the conventionally true values. These components were the beacon rotation relative to the rotation stage movable disk and the supporting base rotation that the rotation stage was placed on. Another factor that could adversely affect the measurement accuracy of both position angles was the mutual tilt of the camera and the beacon, i.e., a mutual roll of these objects around the camera’s optical axis (see Figure 2). Two check measurements were performed with the aim of quantitatively assessing the mentioned inaccuracies.

The first check measurement helped to assess the influence of the undetermined components of the azimuth. The beacon was set with a maximum azimuth deviation of approximately 1.0° towards the rotation stage movable disk. The measurements were performed for two nominal elevations of 0 and 35°. For each elevation, the supporting base was set for initial azimuths of −6.2, 0, and 6.2°. For each of these azimuths, several conventionally true azimuths were set on the rotation stage. The resulting absolute azimuth deviations, relative to the initial azimuths of the supporting base, did not exceed 0.9 and 0.6° for the nominal elevations of 0 and 35°, respectively. The measurement errors were comparable to the errors obtained from the basic experiments. It is clear that for practical use of this method, a firm fixation between the observed object and the measuring pattern has to be ensured.

The influence of the mutual tilt between the camera and the beacon was also measured at nominal elevations of 0 and 35°. For each elevation, their mutual tilt γ_r_ was set at −1.1, 0.0, and 0.9°. The error span and the maximum measurement errors are provided in Table 6. It shows that the mutual tilt between the camera and the beacon influences the measurement error magnitude. However, for the selected relatively small tilts, it demonstrated itself only by a small accuracy worsening. In some cases, the errors were even smaller for the nonzero tilt than for the zero tilt. This phenomenon can be explained by the difference between the beacon parameters used in the mathematical model and the actual beacon parameters.

### 4.5. Influence of the Mathematical Model

The errors resulting from determining inaccuracies of the pixel coordinates, as well as the beacon parameter inaccuracies, are also influenced by the mathematical model character, formed by a system of working equations. The equation’s core is represented by the ratios of the distance projections between the reference diodes and the functional diodes and the corresponding distance projections of the diode images. The object distances are expressed by trigonometric functions of the azimuth and elevations. Both the azimuth and the elevation are independent variables that are systematically changed until the required solution, based on the above-mentioned rule, is found (see Section 2). The coefficients and the initial angles in the working equations are taken from the beacon parameters. The differences between the parameters used in the mathematical model and the actual beacon parameters contribute to the systematic errors. These differences burden the measurement accuracy of the individual functional diodes to different extents, depending on the actual position angles. These mentioned parameter differences cause various functional distance differences, for all functional diodes.

Sizes of images are determined from the pixel coordinates of the corresponding diodes. Errors of the pixel coordinate determination play a significant role in the measurement errors of all the position coordinates. Minimum systematic errors are given by the size of one pixel as it determines elementary measurement uncertainty that cannot be reduced. A manifestation of this uncertainty depends on the picture size, beacon parameters, and measured position angles.

**Figure 7:**
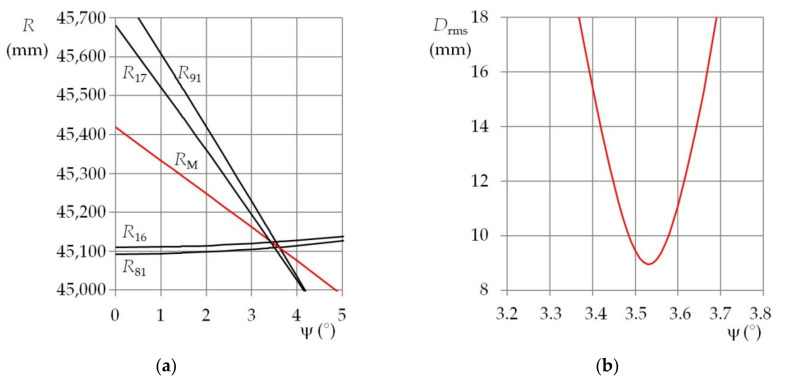
(**a**) Plots of the functional distances for diodes S_6_, S_7_, S_8_, and S_9_; (**b**) root mean square of the distance deviations for diodes S_6_, S_7_, S_8_, and S_9_; *R*_0_ = 46,820 mm, *f*_L_ = 120 mm, ω_n_ = ψ_n_ = 0°.

**Figure 8:**
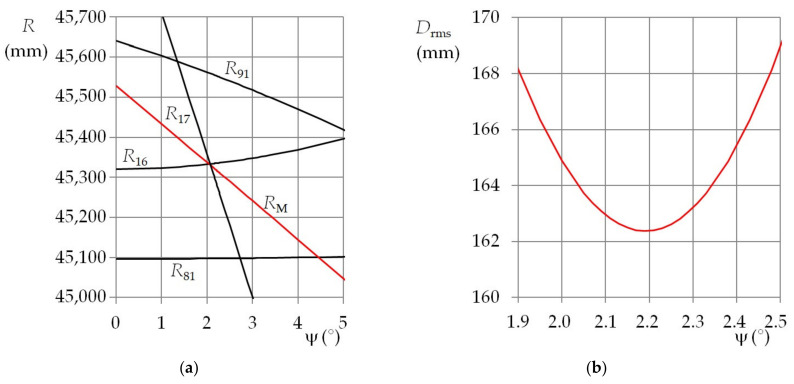
(**a**) Plots of the functional distances for diodes S_6_, S_7_, S_8_, and S_9_; (**b**) root mean square of the distance deviations for diodes S_6_, S_7_, S_8_, and S_9_; *R*_0_ = 46,820 mm, *f*_L_ = 120 mm, ω_n_ = 20°, ψ_n_ = 0°.

This fact is illustrated in Figure 7a and Figure 8a. They show the dependence of the functional distance, for the selected functional diodes, on the elevation that is entered into the mathematical model. Two combinations of the nominal position angles were used. Individual plots show azimuths when the rms minimum of the distance deviation *D*_rms_ was achieved. Due to the above-mentioned errors, the *D*_rms_ minimum had different levels for various combinations of the azimuth and the elevation. In addition, the rate at which the *D*_rms_ was approaching its minimum depended on the actual position angles. These properties are evident in Figure 7b and Figure 8b.

The presented results are from the first experiment (with beacon distance of 46,820 mm and a lens with focal length of 120 mm) for ω_n_ = ψ_n_ = 0° (see Figure 7a) and for ω_n_ = 20° and ψ_n_ = 0° (see Figure 8a). The plots show not only the functional distances *R*_16_, *R*_17_, *R*_81_, and *R*_91_ for the reference diode S_1_ and the functional diodes S_6_, S_7_, S_8_, and S_9_, but also the calculated functional distance means *R*_M_. In addition, the plots of the rms of the distance deviations *D*_rms_ for the mentioned diodes and the nominal position angles are shown in Figure 7b and Figure 8b. The functional distances are shown depending on elevation. The azimuth entered into the mathematical model was actually the parameter of the respective functions. The rms of the distance deviations was evaluated during the measurement process. Its only unambiguous minimum was determined using the entered azimuth and its appropriate elevation. By changing the azimuth, the minimum *D*_rms_ was shifted along the elevation axis and changed in size. Generally, a local minimum for a function with two variables was searched for. When the smallest rms was achieved, then the mean of the functional distances, the entered azimuth, and the appropriate elevation were the sought beacon coordinates. 

It is clear from the plots of the functional distances that the measurement errors depend on the combination of the distance, azimuth, and elevation. If the measurements were burdened only with systematic errors, resulting from the incorrect determination of the actual position coordinates, the mean of the errors would be approximately constant. As the level of the random errors depended on the mentioned combination of the measured quantities, the mean errors expressed as a function of the azimuth often showed a certain trend—most often a significant local extreme (see Figure 4, Figure 5 and Figure 6).

This fact is clear from the functional distance plots. Each diode plot line has a different slope. If the slope is not steep, the deviations between the model parameters and the actual beacon parameters are the significant cause of the measurement errors. This applies especially to the beacon opening angle. It can happen that a small deviation of the beacon parameters has to be compensated by azimuths or elevations that differ very much from the actual values. If the slope of the functional distance line is steep, then the pixel coordinate errors will manifest for the image of the corresponding functional diode. As the distances between the images of the corresponding functional diodes and the reference diode are very small, the deviation of one or two pixels can cause big errors.

An important feature of the mathematical model is the fact that the pixel coordinate errors and the differences between parameters of the real beacon and the mathematical model do not have the same influence on the measurement errors. This is true in the whole range of the measured quantities and their combinations. Thus, the adjustment of the measuring system using optimization of the mathematical model parameters is possible only for some selected intervals of the measured quantities and their combinations. This mathematical model feature was especially apparent for the beacon opening angle.

## 5. Mathematical Model Adjustments

The influence of the beacon and camera parameters on the measurement results can be utilized for the adjustment of the mathematical model that contains all the relevant parameters. In general, it means that the measurement errors can be minimized by changing the system parameters in the mathematical model. As the errors are not linearly dependent on the measured quantities, the adjustment was performed using an optimization of the selected accuracy indicator. This indicator was selected from a group of results containing several combinations of the measured variables. The results were then compared with conventionally true values of the measured beacon position coordinates. Their high determining accuracy is the necessary precondition for successful adjustment. The measuring system can be adjusted using either the focal length or the beacon opening angle or the beacon base that manifests itself similarly to the focal length.

### 5.1. Mathematical Model Adjustment Using the Model Focal Length

Distance measurement errors can be reduced by selecting a suitable focal length *f*_M_ for the model (hereinafter *model focal length*). The position angle errors were affected by the focal length *f*_M_ only slightly. The focal lengths *f*_M_ entered into the model were 125.00 and 26.31 mm, where the actual real focal lengths *f*_L_ were 120 and 25 mm, respectively. The adjustment performed with the model focal length *f*_M_ is illustrated in Table 7 for *f*_L_ = 25 mm, *R*_0_ = 46,730 mm, and ψ_n_ = 35°. It is clear that small changes in the model focal length significantly affect the distance percentage errors.

Since the measured position angles remain almost constant while the model focal length is changing, the focal length *f*_M_ can be optimized by finding the minimum of the biggest distance percentage error for a random combination of the position angles in the provided beacon–camera configuration. The optimal focal lengths *f*_M_ were found for both beacon distances, performed with a lens with a focal length of 25 mm, and for two beacon elevations. Table 8 shows distance percentage errors for the optimal model focal length *f*_Mo_. Table 9 shows the percentage errors for both beacon distances and for two model focal lengths *f*_M13_ and *f*_M46_, which were determined as the optimal lengths for the elevation of 5°. From the comparison of the individual optimal focal lengths and the corresponding distance percentage errors, it is clear that the utilization of only one model focal length can be regarded as acceptable, within the selected intervals of the measured distances and angles.

### 5.2. Mathematical Model Adjustment Using the Beacon Opening Angle

Adjustment of the mathematical model, by entering the beacon opening angle β_M_ into the model (hereinafter *model opening angle*), consists of position angle error evaluation for different angles β_M_. As an example, Table 10 shows the results for several angles β_M_ and two combinations of nominal azimuth and elevation, [ω_n_; ψ_n_] (°). These combinations were [0; 20] and [20; 20]. Results from the first series of the first experiment were used; the beacon distance and lens focal length were 46,820 and 120 mm, respectively.

For the model opening angles β_M_ (°) ∈ 〈56, 64〉 with the step of 0.5°, the azimuth and elevation errors were evaluated. For every β_M_, the root mean square of the position angle error *PA*_rms_ was determined according to the following formula *PA*_rms_ = ((Δω)^2^ + (Δψ)^2^)^0.5^/2. Model opening angle β_M_ is considered optimal when the *PA*_rms_ is minimum. If the *PA*_rms_ is minimum for more different angles, then the beacon distance percentage error can be taken into consideration.

The model opening angle affects all the measured quantities. It can potentially be used to optimize the measurement of the position angles regardless of their distance errors. Distance errors can be minimized separately by optimizing the model focal length as all the model working equations are linearly dependent on the focal length. However, the adjustment of the mathematical model with the opening angle is not suitable as the optimum opening angles vary significantly with different values and combinations of the measured position angles (see Table 10). In the presented cases, the optimal angles were 63.0° for [0; 20] and 59.5° for [20; 20].

## 6. Conclusions

The results of the performed experiments show that the presented simultaneous passive method is usable not only for two coordinates, but also for three position beacon coordinates. In both cases, the mathematical model was based on lens equations of the lines connecting the individual functional diodes with the reference diode. Their functional distances were derived from these equations, depending on one or two beacon position angles. The task was solved numerically according to the rms of the difference between the individual functional distances and the mean of the functional distances. When the minimum of this rms was reached, then, the mean of the functional distances and the substituted values of the position angles were taken as the required results. The method can be used in practice to determine the mutual position of the beacon and camera with sufficient accuracy not only in the 2D plane, but also in 3D. The measurement accuracy was limited mainly by the camera resolution. In addition, the deviations between the real beacon parameters and the mathematical model parameters also have some negative influence on the measurement errors.

The particular reached measurement accuracy for the selected configurations was comparable between the individual experiments. The distance percentage errors were in the order of tenths of a percent. The mean errors and standard deviations of the azimuth and elevation errors were in order of tenths of an angular degree. An increase in the absolute mean azimuth errors above one degree was caused by systematic errors as a consequence of the undetermined components of azimuths (see Section 4.4).

The measuring system can be adjusted by a suitable selection of some mathematical model parameters. The effects of the model focal length *f*_M_ and the model opening angle β_M_ were verified. The accuracy of the beacon distance measurement can be favorably affected by the focal length *f*_M_ entered into the mathematical model. Thus, it is possible to effectively reduce the distance percentage errors and to find the optimal focal length *f*_Mo_, usable within all the intervals of the individual measured quantities, while the influence on the azimuth and elevation remains negligible. On the contrary, the influence of the angle β_M_ is reflected in all the measured coordinates. Thus, the angle β_M_ is potentially suitable for the azimuth and elevation; however, its utilization in practice is not suitable as its optimal value depends on the current β_M_ value and on the position angle combinations.

Analogously, the minimum and the maximum measured beacon distances were affected by the beacon size as much as by the focal length. The layout of the functional diodes, and their mutual position with the reference diode, had influence mainly on the span of the measured position angles. All equations of the mathematical model could not be used when some diodes appeared in one line and were not distinguishable or visible. This situation occurred for large beacon distances or when the position angles exceeded their limits ω_l_ and ψ_l_. The limits are clearly provided not only by the beacon layout but also, in general, by both position angles; for ψ = 0° the ω_l_ = β, and for ω = 0° the ψ_l_ = 90°.

The option to use this method, but with a smaller number of diodes, was verified too. Only five diodes were used in this model. The diode S_1_ was used as a reference diode, and the diodes S_6_, S_7_, S_8_, and S_9_ were tested as the functional diodes (see Figure 1). It is clear from the results that the method was also suitable. However, it was necessary to select only diodes for which the azimuth and the elevation had opposite manifestations. In other words, the line projections between the diodes had to extend for some functional diodes and shorten for others. The resultant errors were comparable with errors listed in Table 5, Table 6 and Table 7.

The presented method is potentially suitable for measuring the position of a random known object. Both the functional and the reference points have to be defined on the surface of this object. In an ideal case, all of these points are visible in the camera pictures. Their images have to be clearly visible so that the distances between the reference point and the individual functional points in the pictures are measurable. Analogously, with this purpose-built beacon, the mathematical model of the object of interest has to contain equations including the line projections (between the reference point and the individual functional points) in the corresponding plane and the line images in the plane of the camera detector.

## Figures and Tables

**Table 1:** Nominal, conventionally true, and measured beacon position coordinates; first test.

Series		ω_n_ (°)						
		−3	0	3	10	20	35	46
1	*R*_m_ (mm)	46,407	46,457	46,486	46,588	46,708	46,875	46,858
	ψ_0_ (°)	21.6	21.6	21.6	21.5	21.5	21.3	21.2
	ψ_m_ (°)	21.3	21.5	21.5	21.3	20.8	20.5	21.1
	ω_0_ (°)	−3.3	0	3.5	10.3	19.8	35.0	45.8
	ω_m_ (°)	−4.5	−1.1	2.2	9.1	18.7	33.8	44.8
2	*R*_m_ (mm)	46,402	46,472	46,474	46,553	46,733	46,974	46,931
	ψ_0_ (°)	21.6	21.6	21.6	21.5	21.5	21.3	21.2
	ψ_m_ (°)	21.4	21.1	21.1	20.8	20.8	20.5	21.1
	ω_0_ (°)	−3.3	0	3.4	10.3	20.0	35.00	46.1
	ω_m_ (°)	−4.5	−1.3	2.2	9.0	18.8	33.7	45.0

**Table 2:** Summary of the distance measurement results.

	ψ_n_ (°)			
	0	5	20	35
	*f*_L_ = 120 mm, *R*_0_ = 46,520 mm, first test			
$\bar{\delta R}\;(\%)$	−0.31	−0.33	−0.43	−0.38
*s*_δ*R*_ (%)	0.21	0.27	0.42	0.76
*F*_δ*R*_ (%)	20/74/100	23/66/100	9/43/94	0/17/66
	*f*_L_ = 25 mm, *R*_0_ = 13,460 mm, second test			
$\bar{\delta R}\;(\%)$	−0.39	−0.67	−0.43	−0.29
*s*_δ*R*_ (%)	0.33	0.33	0.48	0.78
*F*_δ*R*_ (%)	17/66/91	0/34/83	31/57/80	9/66/74
	*f*_L_ = 25 mm, *R*_0_ = 46,728 mm, third test			
$\bar{\delta R}$ (%)	0.58	0.24	0.19	0.26
*s*_δ*R*_ (%)	0.40	0.28	0.44	0.80
*F*_δ*R*_ (%)	6/46/77	26/71/100	11/69/97	9/57/71

$\bar{\delta R}\;(\%)$ is the mean percentage error of the distance. *s*_δ*R*_ (%) is the sample standard deviation of the distance percentage errors. *F*_δ*R*_ (%) is the frequency for absolute distance percentage errors 0.0 to 0.1%/0.0 to 0.5%/0.0 to 1.0%.

**Table 3:** Summary of the azimuth measurement results.

	ψ_n_ (°)			
	0	5	20	35
	*f*_L_ = 120 mm, *R*_0_ = 46,520 mm, first test			
$\bar{\Delta \omega }\;({}^{\circ})$	−1.55	−1.92	−1.20	−1.95
*s*_Δ__ω_ (°)	0.38	0.35	0.11	0.36
*F*_Δ__ω_ (%)	20/74/100	23/66/100	9/43/94	0/17/66
	*f*_L_ = 25 mm, *R*_0_ = 13,460 mm, second test			
$\bar{\Delta \omega }$ (°)	−4.1	−1.6	−1.8	−2.6
*s*_Δ__ω_ (°)	0.1	0.2	0.3	0.5
*F*_Δ__ω_ (%)	100/100/100	100/100/100	97/97/100	63/97/100
	*f*_L_ = 25 mm, *R*_0_ = 46,728 mm, third test			
$\bar{\Delta \omega }$ (°)	−2.0	−1.5	−1.8	−1.9
*s*_Δ__ω_ (°)	0.4	0.2	0.4	0.5
*F*_Δ__ω_ (%)	94/97/100	100/100/100	74/89/100	57/100/100

$\bar{\Delta \omega }\;({}^{\circ})$ is the mean error of the azimuth. *s*_Δω_ (°) is the sample standard deviation of the azimuth errors. *F*_Δω_ (%) is the frequency for absolute azimuth errors 0.0 to 0.5°/0.0 to 1.0°/0.0 to 2.0°.

**Table 4:** Summary of the elevation measurement results.

	ψ_n_ (°)			
	0	5	20	35
	*f*_L_ = 120 mm, *R*_0_ = 46,520 mm, first test			
$\bar{\Delta \psi }\;({}^{\circ})$	−0.9	−1.0	−0.4	−0.2
*s*_Δ__ψ_ (°)	0.5	0.4	0.3	0.3
*F*_Δ__ψ_ (%)	20/54/100	9/49/100	57/91/94	77/100/100
	*f*_L_ = 25 mm, *R*_0_ = 13,460 mm, second test			
$\bar{\Delta \psi }\;({}^{\circ})$	−0.6	−0.6	−0.4	−0.7
*s*_Δ__ψ_ (°)	0.4	0.4	0.3	0.6
*F*_Δ__ψ_ (%)	43/80/100	37/87/100	54/100/100	54/69/100
	*f*_L_ = 25 mm, *D* = 46,728 mm, third test			
$\bar{\Delta \psi }$ (°)	−0.8	−0.8	−0.3	−0.2
*s*_Δ__ψ_ (°)	0.9	0.5	0.4	0.6
*F*_Δ__ψ_ (%)	34/51/89	31/69/97	74/94/100	49/86/100

$\bar{\Delta \psi }\;({}^{\circ})$ is the mean error of the elevation. *s*_Δψ_ (°) is the sample standard deviation of the elevation errors. *F*_Δψ_ (%) is the frequency for absolute elevation errors 0.0 to 0.5°/0.0 to 1.0°/0.0 to 2.0°.

**Table 5:** Changes of measured angles when changing pixel coordinates.

Diode	*y*_C0_	*z*_C0_	*y*_C0_ + 1	Δω_p1_	Δψ_p1_	*y*_C0_ + 2	Δω_p2_	Δψ_p2_
1	1009	239	1010	0.3	0	1011	0.5	0.1
2	1230	242	1231	0	0.1	1232	0	0.3
3	789	236	790	0.1	−0.3	791	0.1	−0.5
4	1223	688	1224	0.1	−0.1	1225	0.1	−0.1
5	773	679	774	0.1	−0.1	775	0.1	−0.1
6	1578	225	1579	0	0	1580	−0.1	0.2
7	1566	668	1567	0	−0.2	1568	0	−0.4
8	363	202	364	0	−0.1	365	0	−0.2
9	350	649	351	0	0	351	0	0

**Table 6:** Effect of the mutual tilt between the camera and the beacon.

ψ_n_ (°)	γ_r_ (°)			γ_r_ (°)		
	0	−1.1	0.9	0	−1.1	0.9
	**δ*D*_max_ − δ*D*_min_ (%)**			**|δ*D*** **|_max_ (%)**		
0	0.71	0.77	0.83	0.36	0.45	0.53
35	1.94	1.71	1.56	1.37	1.27	1.32
	**Δω_max_ − Δω_min_ (°)**			**|Δω** **|_max_ (°)**		
0	0.58	0.74	0.63	0.38	0.74	0.60
35	3.30	3.40	3.29	1.82	1.88	1.87
	**Δ** **ψ_max_ − Δ** **ψ_min_ (°)**			**|Δ** **ψ|_max_ (°)**		
0	1.7	2.7	1.4	1.2	1.5	1.4
35	1.2	2.1	0.8	0.9	1.4	1.0

**Table 7:** Distance percentage errors δ*R* (%) for different model focal lengths.

	ω_n_ (°)						
	−3	0	3	10	20	35	46
δ*R* (%) for *f*_M_ = 26.31 mm	−1.23	−1.11	−0.88	−0.75	−0.36	0.59	0.77
δ*R* (%) for *f*_M_ = 26.00 mm	−1.55	−1.21	−1.31	−0.69	−0.92	0.16	0.32
δ*R* (%) for *f*_M_ = 26.50 mm	0.34	0.70	0.59	1.20	0.99	2.05	2.26

**Table 8:** Distance percentage errors δ*R* (%) for the optimal model focal length.

	ω_n_ (°)						
	−3	0	3	10	20	35	46
	*f*_L_ = 25 mm, *R*_0_ = 13,460 mm, ψ_n_ = 5°						
δ*R* (%) for *f*_Mo_ = *f*_M13_ = 26.15 mm ^1^	−0.40	−0.20	−0.10	0.03	0.04	0.35	0.42
	*f*_L_ = 25 mm, *R*_0_ = 13,460 mm, ψ_n_ = 35°						
δ*R* (%) for *f*_Mo_ = 26.17 mm	−1.04	−0.85	−0.86	−0.62	−0.12	0.76	1.06
	*f*_L_ = 25 mm, *R*_0_ = 46,730 m, ψ_n_ = 5°						
δ*R* (%) for *f*_Mo_ = *f*_M46_ = 26.19 mm ^2^	−0.37	−0.10	−0.07	−0.06	−0.17	−0.07	0.39
	*f*_L_ = 25 mm, *R*_0_ = 46,730 m, ψ_n_ = 35°						
δ*R* (%) for *f*_Mo_ = 26.16 mm	−0.95	−0.60	−0.70	−0.10	−0.31	0.74	0.94

^1^ The model focal length, which was determined as optimal for the beacon distance of 13,460 mm. ^2^ The model focal length, which was determined as optimal for the beacon distance of 46,730 mm.

**Table 9:** Errors for various optimal model focal lengths.

	ω_n_ (°)						
	−3	0	3	10	20	35	46
	*f*_L_ = 25 mm, *R*_0_ = 13,460 mm, ψ_n_ = 5°						
δ*R* (%) for *f*_M13_ = 26.15 mm ^1^	−0.40	−0.20	−0.10	0.03	0.04	0.35	0.42
δ*R* (%) for *f*_M46_ = 26.19 mm ^2^	−0.24	−0.05	0.06	0.20	0.20	0.50	0.60
	*f*_L_ = 25 mm, *R*_0_ = 46,730 mm, ψ_n_ = 5°						
δ*R* (%) for *f*_M13_ = 26.15 mm ^1^	−0.52	−0.26	−0.22	0.20	0.30	0.20	0.20
δ*R* (%) for *f*_M46_ = 26.19 mm ^2^	−0.37	−0.10	−0.07	0.06	0.17	0.07	0.39

^1^ The model focal length, which was determined as optimal for the beacon distance of 13,460 mm. ^2^ The model focal length, which was determined as optimal for the beacon distance of 46,730 mm.

**Table 10:** Optimization of the model opening angle.

	β_M_ (°)												
	56.0	56.5	57.0	57.5	58.0	59.0	59.5	60.0	60.5	61.0	62.0	63.0	64.0
	ω_n_ = 0°, ψ_n_ = 20°												
Δω (°)	−1.11	−1.11	−1.11	−1.11	−1.11	−1.21	−1.21	−1.21	−1.21	−1.21	−1.31	−1.31	−1.41
Δψ (°)	1.45	1.45	1.35	1.25	1.25	1.15	1.05	1.05	0.95	0.95	0.85	0.75	0.65
*PA*_rms_ (°)	1.29	1.29	1.24	1.18	1.18	1.18	1.13	1.13	1.09	1.09	1.10	1.07	1.10
	ω_n_ = 20°, ψ_n_ = 20°												
Δω (°)	−1.69	−1.39	−1.19	−0.99	−0.69	−0.19	0.01	0.31	0.51	0.81	1.41	1.91	2.51
Δψ (°)	0.20	0.20	0.30	0.30	0.30	0.40	0.40	0.50	0.50	0.50	0.60	0.70	0.80
*PA*_rms_ (°)	1.20	0.99	0.87	0.73	0.53	0.31	0.28	0.42	0.51	0.67	1.08	1.44	1.86

## Data Availability

Not applicable.
